# A model linking emotional dysregulation in neurodivergent people to the proprioceptive impact of joint hypermobility

**DOI:** 10.1098/rstb.2023.0247

**Published:** 2024-08-26

**Authors:** Jessica A. Eccles, Lisa Quadt, Sarah N. Garfinkel, Hugo D. Critchley

**Affiliations:** ^1^ Department of Clinical Neuroscience, Brighton and Sussex Medical School, Falmer BN1 9RY, UK; ^2^ Sussex Neurodevelopmental Service, Sussex Partnership NHS Foundation Trust, West Sussex, BN2 3EW, UK; ^3^ Institute of Cognitive Neuroscience, University College London, London WC1N 3AZ, UK

**Keywords:** hypermobility, neurodivergence, predictive processing, proprioception, emotional dysregulation

## Abstract

Emotional feelings are putatively ascribed to central representation of bodily states in the context of expectation and uncertainty in both internal state and external world. Neurodivergent people are more likely to experience co-occurring mental health challenges, although mechanistic insights underpinning this association are scarce. We therefore undertook a study to test whether imprecise processing of proprioceptive error signals may underlie the connection between neurodivergence and emotional dysregulation. In a cohort of people with complex chronic conditions, including chronic pain/fatigue, and complex trauma, and in a comparison group, we assessed presence of neurodivergence, variant connective tissue manifested through joint hypermobility, and emotional dysregulation. We present a data-informed conceptual model showing that variant connective tissue determines whether proprioceptive surprise is linked with emotional dysregulation in neurodivergent individuals. We suggest that future research in this area may have important clinical implications for the interaction of mental and physical wellbeing in neurodivergent people.

This article is part of the theme issue ‘Sensing and feeling: an integrative approach to sensory processing and emotional experience’.

## 1. Introduction

### (a) Embodied feeling

Influential theories link emotions to the state of the body: feeling and sensing are inherently coupled. Basic and clinical neuroscience, including, for example, that led by Damasio and colleagues (e.g. [1]) has helped guide the neurophysiological reformulation of seminal propositions of William James and Carl Lange (e.g. [2]). Here, affective feelings and associated motivational behaviours are linked to changing interoceptive representations that interact with sensed changes in exteroceptive context. Moreover, emotional feelings often have visceral and/or muscular sensory signatures, e.g. ‘heart racing, ‘stomach churning’, ‘blood draining’ and ‘hot-headed or ‘tense’. Uncertainty is an important driver of salience: interoceptive changes (e.g. enhanced state of cardiovascular arousal) may trigger and intensify the emotional feelings of anxiety in the absence of a plausible proximate cause (e.g. effortful action) [3,4]. Predictive coding models argue that interoceptive surprise (i.e. signalling unexpected bodily responses), represented as prediction error signals, motivate emotional learning and behaviour through the intensification of physiological feelings, i.e. affective experience [5]. By extension, individual differences in the reliability and strength of bodily signalling will determine emotional impact ascribed to bodily prediction errors [6] such that precision of interoceptive regulation is a contextual determinant of sensing and feeling. In this paper, this proposal is empirically examined with reference to neurodiversity, notably neurodivergent emotional experiences, and to joint hypermobility, a common variant in bodily constitution related to difference in connective tissue structure.

### (b) Neurodivergent emotional experience

The term ‘neurodivergence’ supersedes a deficit-based medical model [7] of neurodevelopmental traits and conditions, including autism and attention deficit hyperactivity disorder (ADHD), to acknowledge a variety of characteristics and strengths [8]. Here, we attempt to align our language with guidance from the neurodiversity movement [9]. However, neurodivergent people experience a variety of mental health concerns [10,11]. In neurodivergent people, the concept of bodily prediction errors has particular explanatory value in understanding the heightened expression of anxiety [12] and is a target for effective interventions [13]. While autonomic and interoceptive aspects of physiological regulation remain an important focus for understanding affective experience and emotional vulnerability in neurodivergent individuals, it is recognized that developmental differences in proprioception (for example, as expressed as dyspraxia) are at the interface between the internal self and external world, and are also commonly associated with neurodivergence, for example within the ESSENCE framework [14], and are an area of growing research (e.g. [15]).

### (c) Role of proprioception

The relationship between proprioception (sense of the body's position in space) and emotion has been largely under-explored. This contrasts with interoception, where viscerosensory signalling of dyshomeostatic states such as cold and hunger is recognized to underpin motivational and ultimately emotional feelings and behaviour. Nevertheless, the emotion–proprioception relationship appears bidirectional: emotions (as different states of action-readiness) influence posture [16], such that one can view posture as a channel of emotional communication [17]. Conversely, posture will influence the perceptual processing of external information [18]. Moreover, vestibular signals exert a rapid and strong influence on autonomic responses [19], and high rates of anxiety and depression are observed in patients with vestibular disorders (e.g. [20]). Although the boundaries between proprioception and vestibular processing may be under debate, they are closely linked [21], and despite such circumstantial associations, the role of proprioception, in particular the impact of proprioceptive imprecision, on emotional feeling states has hitherto been poorly conceptualized.

### (d) Variant connective tissue: a potential exemplar of embodied sensing and feeling

There is growing interest in variant connective tissue structure (often manifest as joint hypermobility) as a potential model to explore brain–body interactions [22,23]. Joint hypermobility (increased flexibility and range of joint movement) arises from differences in the elasticity compliance of joint ligaments and tendons and is the most describable feature of systemic differences in connective tissue (and constituents such as collagen and extracellular matrix). Hypermobility is typically more common in females and declines with age. It is not necessarily a medical problem, but certain clinical phenotypes, notably hypermobile Ehlers–Danlos syndrome (hEDS; previously known as EDS hypermobility type/EDS type-III), and hypermobility spectrum disorder (HSD) are associated with clinically significant medical issues, including dysautonomia [24]. Prevalence rates vary according to ethnicity and sex worldwide (e.g. [25]), but around 20% of the UK population are likely hypermobile (generalized joint hypermobility) [26]. Joint hypermobility is associated with interoceptive and autonomic differences, and is linked to differences in expression of anxiety and related emotional states across diagnostic groups [27]. In hypermobile individuals, corresponding differences in neural function and structure are observed in brain regions that support both emotional experience (amygdala and insular cortices) and proprioception (inferior parietal lobule volume) [23,28,29].

Hypermobility is associated with developmental proprioceptive differences [30,31]. Altered sensation accompanying exaggerated articular range across joints is linked to developmental dyspraxia and related issues with coordination, including differences in gait and the propensity to develop joint pathology (osteoarthritis) through increased wear. Hypermobility thus may predispose to proprioceptive imprecision, though this may be mitigated by muscle strengthening and movement training, for example in dance, gymnastics and sports that exploit aesthetic and functional advantages of being hypermobile. We hypothesize that imprecise (hence poorly predicted) proprioceptive signals enhance the generation of bodily prediction errors and their translation into emotional feelings and affective phenomenology. We explored this relationship in the context of neurodivergence where differences in the interaction between physical and psychological states are likely to have been established developmentally.

### (e) Testing the hypothesis

We quantified features of variant connective tissue, proprioceptive sensory imprecision (manifest as proprioceptive hyper/hyposensitivity), emotional regulation and neurodivergence. Our objective was to provide empirical evidence for a conceptual model in which proprioceptive surprise is proposed to be a determinant of differences in affective regulation in neurodivergent individuals through constitutional variation in brain and body. We tested the hypothesis using mediation and moderated mediation models (Hayes Conditional Process Analysis). In such analyses a mediator may explain the process of a relationship between linked variables (e.g. proprioceptive surprise may link neurodivergence and emotion regulation) and a moderator (e.g. presence of joint hypermobility) may affect the strength or direction of that relationship.

## 2. Method

### (a) Study design and participants

This pragmatic analysis is conducted on data collected as part of a broader investigation (‘NeuroCats’ study). This original study examined the relationship between complex chronic conditions (CCC, operationalized as diagnoses of chronic pain, fatigue and complex trauma), neurodivergent characteristics, and hypermobility. This study included both adults with complex chronic conditions (CCCs) and a non-clinical matched comparison group (both groups *n* = 91, total *n* = 182). Complex chronic condition participants, with one or more diagnosis of borderline personality disorder/emotionally unstable personality disorder (BPD/EUPD), complex post-traumatic stress disorder (C-PTSD), chronic pain (e.g. fibromyalgia), and/or chronic fatigue (e.g. ME/CFS), were recruited via social media, community and third-sector organizations. Comparison participants without a CCC diagnosis were recruited via the online platform *Prolific* (and excluded if they rated pain or fatigue greater than 30/100 on screening visual analogue scales or reported neurodegenerative or psychiatric conditions other than anxiety or depression). For the NeuroCats survey, participants were matched by sex assigned at birth (since CCCs are more common in females), by ethnicity (since joint hypermobility is overrepresented in some ethnicities), and by level of education. The presence of complex chronic conditions was not the subject of analysis for this study, and in this analysis based on likely neurodivergence (see below for thresholds), there were no reported differences in age or sex between groups (see Results). The study was approved by Brighton and Sussex Medical School Research and Governance Ethics Committee (ER/BSMS9B02/2).

### (b) Outcomes

Participants provided demographic information and completed a set of questionnaires. These included the 80-item Ritvo Asperger Autism Diagnostic Scale (RAADS) [32] to index autistic traits, and the Wender Utah Rating Scale (WURS) [33] to index ADHD traits. Those scoring above established thresholds on the RAADS (≥65) and/or WURS (≥46) screening questions were classified as neurodivergent. Participants also completed a five-item joint hypermobility questionnaire (see box 1). A score ≥2 indicated likely presence of significant joint hypermobility [34]. A total proprioceptive symptom sub-score (encompassing self-rated hypo- and hyper-sensitivities) was derived from completion of the Glasgow Sensory Questionnaire [35] and used as our measure of degree of proprioceptive imprecision (see box 2). Affective dysregulation was operationalized as both the total score on the Dissociative Experiences Scale_II (DES-II) and additionally binarized according to established thresholds (≥30) [36].

Box 1. **Hypermobility screening measure [34]**

**The Five-Part Hakim and Grahame Questionnaire (5PQ) for defining generalized joint hypermobility**

Can you now (or could you ever) place your hands flat on the floor without bending your knees?

Can you now (or could you ever) bend your thumb to touch your forearm?

As a child did you amuse your friends by contorting your body into strange shapes OR could you do the splits?

As a child or teenager did your shoulder or kneecap dislocate on more than one occasion?

Do you consider yourself double-jointed?

**Endorsement of two or more questions suggests generalized joint hypermobility.**

Box 2. **Proprioceptive domains of Glasgow Sensory Questionnaire [35]**

**Proprioceptive sub-scale of Glasgow Sensory Questionnaire**

Do you find it difficult to manipulate your hands when completing a delicate task (for example, picking up small objects or transferring objects from one hand to the other)? (Q3)

Do you stand very close (for example, less than 1 metre/3 feet away) or very far (for example, more than 3 metres/9 feet away) when you are talking to someone? (Q5)

Do you find that you are unaware of your body's signals (for example, don't often feel hungry/tired/thirsty)? (Q29)

Do you find that you position your body in a way that is different to most people (for example, lie on your back on a sofa with your legs straight up in the air at a 90 degree angle)? (Q37)

Do you find it difficult to tie your shoelaces or button up your clothes? (Q38)

Do you like to wear something/hold something (for example, a hat or a pencil) so that you know where your body ‘ends’? (Q41)

### (c) Statistical analyses

Analyses were conducted using IBM SPSS version 29 incorporating the PROCESS Toolbox version 4.1. Correlations were analysed using Spearman rank, with a Bonferroni correction for multiple comparisons changing the threshold for statistical significance to 0.05/4 = 0.013. Initial separate mediation models (PROCESS model 4), conducted and finally integrated into moderated mediation analyses (PROCESS model 7), were carried out according to the method of Hayes [37]. This allows inference of conditional processes if bootstrapped confidence intervals of the indirect effect and index of moderated mediation (*n* = 5000 in this analysis) do not include zero.

### (d) Patient and public involvement

This study was motivated by experiences described by members of our lived experience advisory forums. Members of the forum guided the design of the original NeuroCats study and assisted in the dissemination of the study's original findings. They remain engaged to help disseminate the summarized findings of this analysis to relevant communities, including support groups. The authorship team includes neurodivergent individuals and those with lived experience of joint hypermobility.

## 3. Results

Ages ranged from 18 to 75, mean age = 39.14, s.d. = 12.68. Across the whole purposively sampled group (*n* = 91 comparison, *n* = 91 CCC), 91 (50%) were hypermobile, 82 (45%) scored above screening threshold for suspected neurodivergence (i.e. scoring above screening threshold on either WURS or RAADS) and 44 (24%) scored above threshold on DES-II. There were no significant differences in age or sex assigned at birth between those operationalized as neurodivergent (mean age 39.55 years, s.d. 12.90, 91% assigned female at birth) or not (mean age 38.63 years, s.d. 12.47, 94% assigned female at birth). Those scoring above threshold on DES-II were younger (mean age 34.48 years, s.d. 10.36) than those below threshold (mean age 40.76 years, s.d. 12.97, *t* = 2.93, d.f = 179, *p* = 0.004) but there were no statistically significant differences in sex assigned at birth (95% and 91% assigned female at birth, respectively).

All four scale variables (number of neurodivergent characteristics, number of joint hypermobility features, degree of emotion dysregulation and proprioceptive surprise) were all found to be significantly correlated with each other (tables 1 and 2, *p* ≤ 0.001), with at least median effect size (all *r* ≥ 0.3) before conditional process analysis was carried out (table 3).

**Table 1.** Participant characteristics: demographic details.

participant characteristics	*n*	%
**sex assigned at birth**		
female	168	87.9
male	14	7.7
**gender identity**		
female	160	87.9
male	15	8.2
non-binary/gender non-conforming	6	3.3
prefer not to say	1	0.5
**education**^a^		
GCSE or similar	15	8.2
A-levels or similar	36	19.8
attended college, no degree	26	14.3
undergraduate degree	60	33
graduate degree	45	24.7
**ethnicity**		
Black	3	1.6
Mixed	3	1.6
White	175	96.2
prefer not to say	1	0.5

^a^Based on UK education system.

**Table 2.** Characteristics of the clinical sample (CCC dataset).

clinical diagnosis	*n*	%
borderline personality disorder (BPD)	17	18.7
complex post-traumatic stress disorder (C-PTSD)	19	20.9
fibromyalgia	38	41.8
ME/CFS	33	36.3
other chronic pain/fatigue diagnosis	53	58.2
both BPD or C-PTSD and chronic pain/fatigue diagnoses	39	42.9

**Table 3.** Correlation matrix for Spearman’s *ρ* in *N* = 182 participants demonstrating significant inter-relationships between hypermobility (Five-Part Hakim and Grahame Questionnaire, 5PQ), emotion dysregulation (Dissociative Experiences Scale_II, DES-II), neurodivergent characteristics (compound *z-*score of Wender Utah Rating Scale (WURS) and Ritvo Asperger and Autism Diagnostic Scale (RAADS)) and proprioceptive surprise (derived from the Glasgow Senory Questionnaire, GSQ). 'For all values of *ρ*, significance (2-tailed) *p* < 0.001.

		hypermobility score	emotion dysregulation	neurodivergent characteristics	proprioceptive surprise
	hypermobility score				
	emotion dysregulation	0.375			
	neurodivergent characteristics	0.496	0.772		
	proprioceptive surprise	0.545	0.694	0.826	

### (a) Mediation model 1. Exploring the relationship between hypermobility and emotion through proprioception

**Figure 1.**
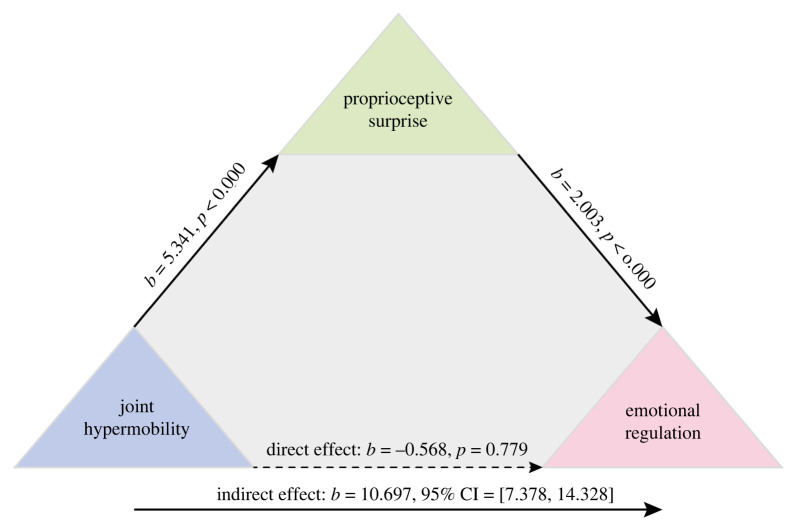
Mediation model linking hypermobility to emotion through proprioception.

In model 1 (figure 1), the presence of hypermobility (*X*) was found to be linked to emotion regulation scale (*Y*) through proprioceptive surprise (*M*) (estimate of indirect effect of *X* on *Y* through *M*: 10.70, bootstrapped 95% CI 7.38–14.32). This effect remained when emotion regulation was binarized (estimate of indirect effect of *X* on *Y* through *M*: 1.37, bootstrapped 95% CI 0.09–2.06).

### (b) Mediation model 2. Exploring the relationship between neurodivergence and emotion through proprioception

**Figure 2.**
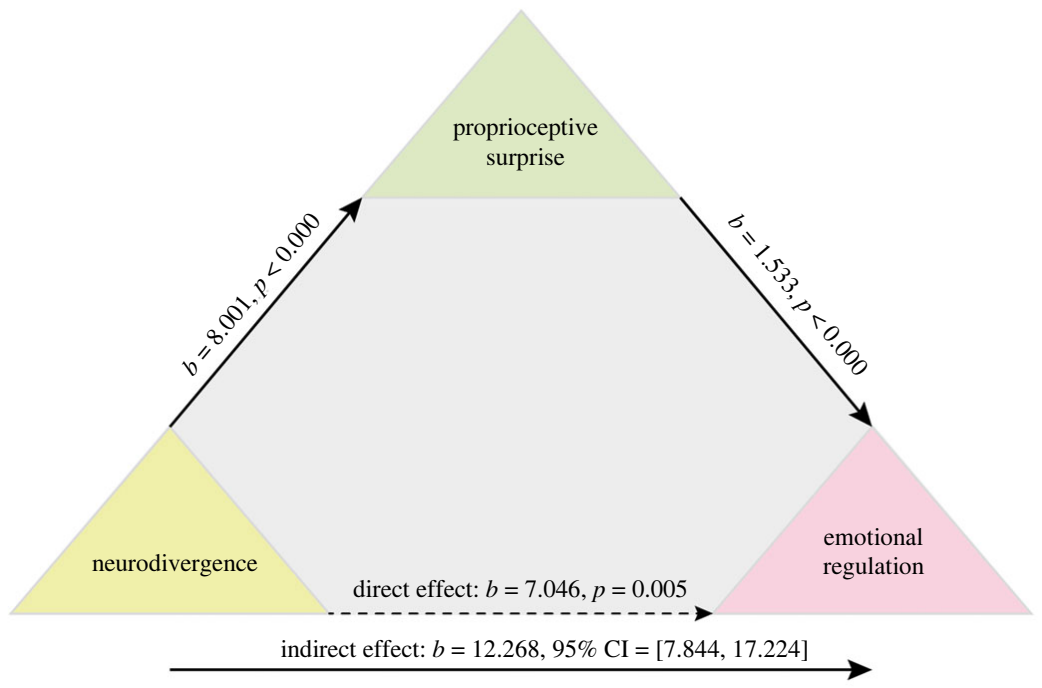
Mediation model linking neurodivergence to emotion through proprioception.

In model 2 (figure 2), the presence of likely neurodivergence (*X*) was found to be linked to emotion regulation (*Y*) through proprioceptive surprise (*M*) (estimate of indirect effect of *X* on *Y* through *M*: 12.27, bootstrapped 95% CI 7.84–17.22). This effect remained when emotion regulation was binarized (estimate of indirect effect of *X* on *Y* through *M*: 1.31, bootstrapped 95% CI 0.60–2.25).

### (c) Integrated model. Combining the models to characterize the relationship between neurodivergence, emotion, proprioception and hypermobility—a moderated mediation model

**Figure 3.**
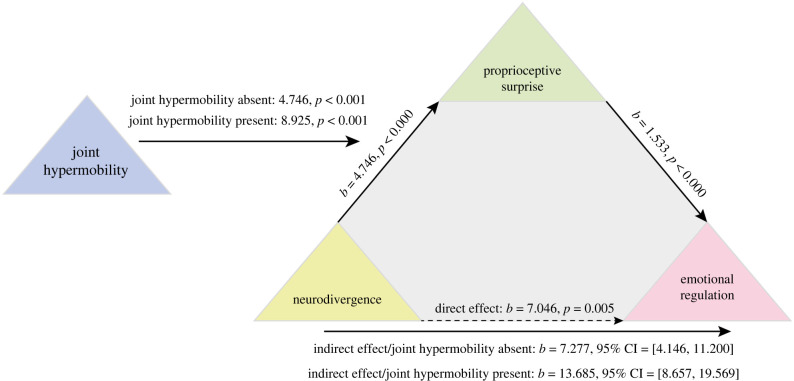
Integrated moderated mediation model demonstrating moderating role of hypermobility on the mediating effects of proprioception on the relationship between neurodivergence and emotion regulation.

In the final integrated model (model 3, figure 3), the presence of hypermobility (*W*) moderated the path linking likely neurodivergence (*X*) to emotion regulation (*Y*) through proprioceptive surprise (*M*) (index of moderated mediation: 6.41, bootstrapped 95% CI 2.51–11.17). This effect remained when emotion regulation was binarized (index of moderated mediation: 0.69, bootstrapped 95% CI 0.21–1.41).

## 4. Discussion

We present for the first time to our knowledge an *in vivo* data-informed conceptual model of how variant connective tissue (hypermobility) influences whether proprioceptive differences (operationalized as proprioceptive surprise) lead to emotion regulation difficulties in neurodivergent people. Our study provides initial data that will help to substantiate and extend existing theoretical proposals (e.g. [1,38]) that suggest that motor behaviour (i.e. the active regulation of bodily position and action) is recruited to regulate feelings and sensing through impact on proprioception and interoception [39]. Our empirically informed model sits alongside and will help inform the broader computational framework of ‘active inference’ in which predictive coding of sensory information is shaped by actions to enable strategic sampling and control of the source of such information, to minimize prediction error [40]. Our study also serves to highlight the often-overlooked potential clinical import of hypermobile and neurodivergent characteristics. In practice, hypermobility and neurodivergence are both frequently missed or overlooked and may be more prevalent than current estimates suggest. Both groups experience delays in diagnosis/recognition, trauma, and difficult medical encounters [41,42]. We suggest increased recognition of the inter-connection between these conditions [23] will prove important in order to improve outcomes and wellbeing across mental and physical health domains.

To our knowledge no empirical work has previously linked these interconnected phenomena. Our use of screening measures rather than diagnostic instruments and reference to a case–control sample that may not be indicative of the general population are acknowledged limitations, alongside a predominately White and female sample. We also acknowledge the inherent difficulties in conducting an analysis on a dataset intended to ask a different research question (association with complex chronic conditions). Additional limitations include not accounting for the role of activity/exercise on either hypermobility or proprioception or the influence of medications. Indeed, we did not include observed measurements of proprioception or include behavioural or computational tasks to probe proprioceptive surprise, which is a significant limitation of this observational study.

However, this approach enabled a large, pragmatic sampling that can be a springboard for further, more detailed, research. Future studies should include appropriate *a priori* matching of characteristics, full diagnostic assessments that can characterize individual features (sub-phenotypes) that potentially may drive this important relationship, and more fine-grained conceptualization of proprioceptive surprise, including behavioural and computational task performance.

Our data, and the mechanistic model that emerges from our finding, have potential importance for therapeutic approaches. Mental health problems cost the UK economy at least £117.9 billion annually. New evidence-based treatments for common mental health conditions are needed and their development is perhaps constrained by adherence to simplified (e.g. cognitive psychological) models. The impact of many interventions, including those currently typically offered though primary care services, is potentially limited: cognitive behavioural therapies (CBT) are challenging for many patients because of issues in discussing emotions (e.g. alexithymia) or in maintaining motivation (e.g. owing to anxiety/depression). Medicines (e.g. antidepressants) are usually helpful but represent blunt instruments, often with unwanted effects muddying their impact on recovery. The fact that ‘one size does not suit all’ is increasingly appreciated. The characterization of dependent interactions between brain and body opens up discussion and possibilities for a much-needed channel for interventional innovation. Understanding the mechanisms of this relationship between sensing and feeling in neurodivergence could potentially inform novel avenues for personalized brain–body-based treatments for common mental health problems, particularly in hypermobile individuals, e.g. treatments that target proprioceptive differences. Proprioception has been shown to be dynamic in hypermobile individuals and has the capacity to be enhanced [43].

This is important because neurodivergent people are often marginalized and can experience significant barriers to health care with adverse outcomes [42,44], loneliness [45] and mental distress [46]. Better understanding of factors underpinning psychological and physical health and flexible integrated strategies to mitigate or manage the emergence of symptoms will hopefully enable neurodivergent people to access the care and resources they need to ensure they thrive [11,47]. We hope to encourage evidence-based interdisciplinary (encompassing body and brain) science and practice in order to achieve this goal.

In summary, we highlight and map the relationship between developmental neurodivergence and constitutional variation in connective tissue to predict proprioceptive mediators of affective experience. Our data support a model wherein representational precision at a sensory level is a determinant of dysregulated emotional feelings. Our empirical data and explanatory model fit within a broader theoretical framework to highlight mechanisms that can improve health and wellbeing, particularly for under-served groups.

## Data Availability

Data can be accessed at https://osf.io/t2d7u/ [48].
